# Magnesium depletion score and gout: insights from NHANES data

**DOI:** 10.3389/fnut.2024.1485578

**Published:** 2024-11-21

**Authors:** Xu Cao, Haixia Feng, Huijie Wang

**Affiliations:** ^1^Department of Endoscopy, Shijiazhuang Traditional Chinese Medicine Hospital, Shijiazhuang, China; ^2^Department of Tuberculosis, Shandong Public Health Clinical Center, Jinan, Shandong, China

**Keywords:** gout, magnesium depletion score, cross-sectional study, National Health and Nutrition Examination Survey, association

## Abstract

**Objectives:**

Gout is associated with hyperuricemia, and serum magnesium levels are negatively correlated with uric acid levels. Magnesium intake is also associated with a reduced risk of hyperuricemia. However, the relationship between the magnesium depletion score (MDS), which represents the systemic magnesium status, and gout is unclear. This study was conducted to investigate the association between MDS and gout as well as explore the impact of dietary magnesium intake on this relationship.

**Methods:**

We analyzed 18,039 adults with gout data who participated in the National Health and Nutrition Examination Survey between 2007 and 2016. Magnesium deficiency status was assessed using the MDS, a comprehensive scoring tool. Considering the possible effects of dietary magnesium intake, weighted multivariable logistic regression and subgroup analyses were used to assess the correlation between MDS and gout.

**Results:**

The overall prevalence of gout among adults in the United States between 2007 and 2016 was 4.7%. After adjusting for confounders, MDS and gout risk showed a significant positive correlation. Individuals with an MDS of 2 and ≥ 3 had higher odds of gout than those with an MDS of 0 (MDS = 2, odds ratio: 1.86 [1.18–2.93], *p* = 0.008; MDS = 3, odds ratio: 2.17 [1.37–3.43], *p* = 0.001; *p* for trend <0.001). Dietary magnesium intake did not moderate the correlation between MDS and gout risk.

**Conclusion:**

A positive correlation exists between magnesium deficiency, as quantified using the MDS, and gout risk among adults in the United States. Additionally, dietary magnesium intake did not alter this association.

## 1 Introduction

Gout is caused by the deposition of monosodium urate crystals in non-joint and joint structures (1). This disease is characterized by severe joint pain, swelling, warmth, and functional impairment, particularly of the metatarsophalangeal joint of the big toe (2). Depending on the population studied and methodologies employed, reports on the global prevalence and incidence of gout vary widely. The prevalence of gout ranges from <1 to 6.8%, and its incidence ranges from 0.58 to 2.89 cases per 1,000 person-year (3). The prevalence and incidence of gout have increased in recent years (4–6), making it an important public health issue.

Magnesium is an essential element in the human body with a crucial role in supporting and maintaining health and life, and should be continuously replenished through dietary intake and water consumption (7). Magnesium is vital for stabilizing the tertiary structure of DNA and RNA; participating in various enzymatic reactions including energy metabolism and protein synthesis; regulating cellular Mg^2+^ handling; and influencing cell signaling and proliferation (8–13). However, previous research primarily focused on the effects of diet and serum magnesium on gout. Serum magnesium levels are negatively correlated with uric acid levels (14), and increased magnesium intake is associated with a reduced risk of hyperuricemia (15). Notably, serum Mg^2+^ levels reflect only approximately 1% of total magnesium content in the body, as most magnesium is stored in the bones, muscles, and soft tissues (7). Moreover, the magnesium content in the body is subject to dynamic fluctuations, which are primarily absorbed through the gastrointestinal tract and excreted via the kidneys (16). Therefore, serum magnesium levels may not accurately represent the overall magnesium status of the body.

The magnesium depletion score (MDS) is a comprehensive scoring tool designed to assess overall magnesium deficiency by aggregating four established risk factors and considering the pathophysiological factors that affect the renal reabsorption capacity, including proton pump inhibitor (PPI) use, diuretic use, alcohol consumption, and renal disease (17). Compared with serum magnesium, the MDS is a more sensitive and reliable measure of an individual’s magnesium deficiency or depletion status (17). Current studies indicate that MDS is significantly associated with various conditions, including systemic inflammation, cardiovascular diseases, renal diseases, metabolic syndrome, hypertension, and diabetes (17–22).

Considering that the association between MDS and gout is unclear, we performed a cross-sectional study using data from the National Health and Nutrition Examination Survey (NHANES). Our aim was to analyze the association between MDS and gout, thereby establishing a stronger link between magnesium status and gout risk, and to explore whether dietary magnesium moderates this relationship.

## 2 Materials and methods

### 2.1 Study population and design

The research data were sourced from the NHANES, conducted by the Centers for Disease Control and Prevention. The NHANES employs a robust, multistage, and stratified sampling method gathering detailed information through interviews and assessments as well as provide valuable insights into demographic and health-related factors of the United States population.

**Figure 1:**
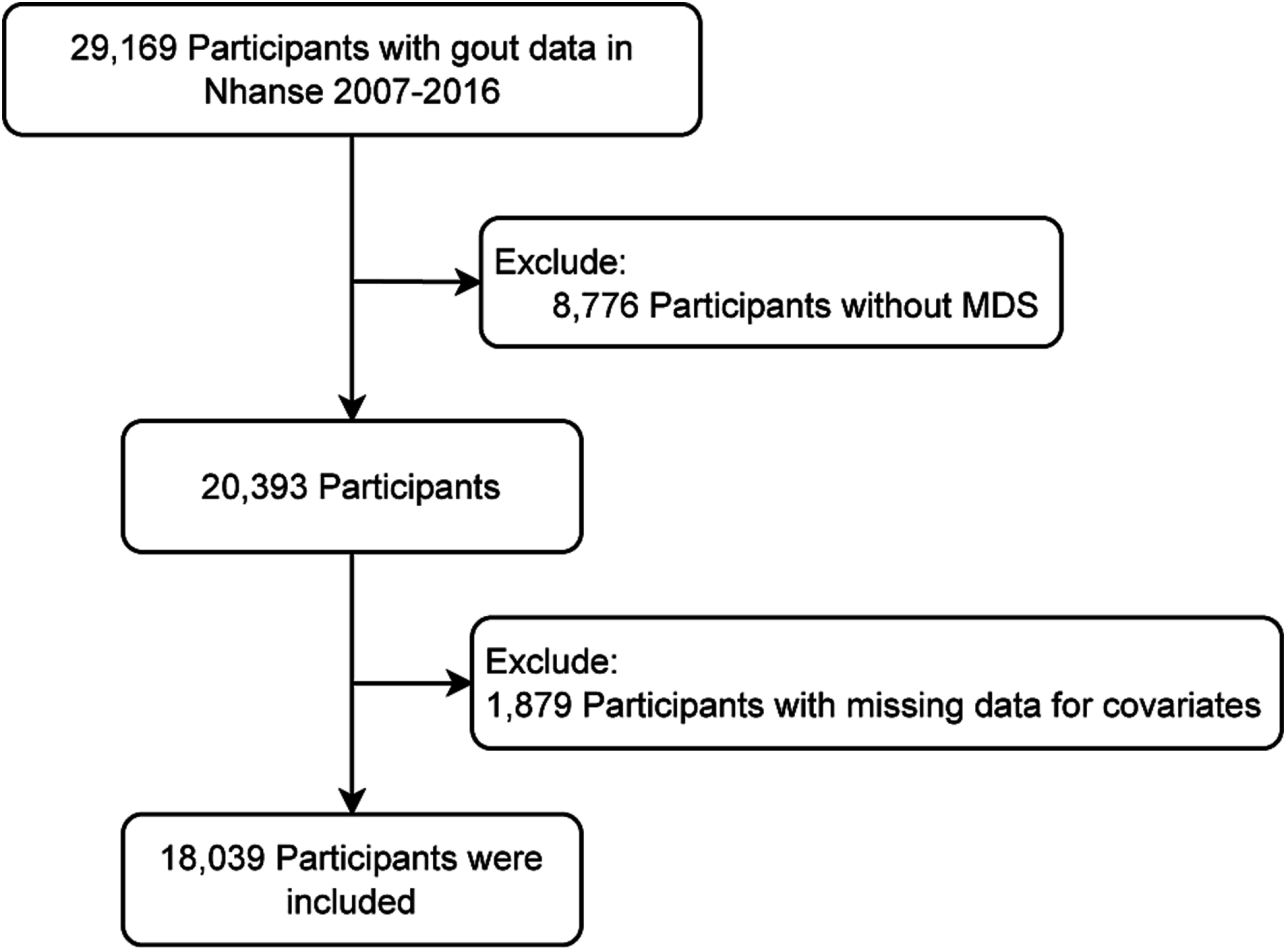
Flowchart of participants.

Based on the availability of MDS and gout information, 29,169 participants were initially included from five cycles of NHANES data (2007–2016). Participants who could not have their MDS determined (*n* = 8,776) or had missing covariates data (*n* = 2,354) were excluded. Ultimately, 18,039 participants were included (Figure 1).

This study was approved by the Research Ethics Review Committee of the National Center for Health Statistics. Each participant provided written informed consent. The NHANES dataset, along with accompanying documentation and agreements, was obtained from the website.

### 2.2 MDS assessment

Magnesium depletion score was calculated by summing the scores of the following entries: one point each for current use of diuretics and PPIs, one point for an estimated glomerular filtration rate of 60–90 mL/(min·1.73 m^2^), two points for an estimated glomerular filtration rate < 60 mL/(min·1.73 m^2^), and one point for heavy drinking (>2 drinks/day for men, >1 drink/day for women) (17). Using the Chronic Kidney Disease (CKD) Epidemiology Collaboration equation, serum creatinine was used to determine the estimated glomerular filtration rate (23). MDS was classified into four groups: MDS = 0, 1, 2, and ≥ 3.

### 2.3 Definition of gout

The presence of gout was determined through self-reported response in a medical condition questionnaire (24).

### 2.4 Covariate assessment

Baseline data included age, sex, race, body mass index (BMI), marital status, smoking status, educational attainment, poverty-to-income ratio, physical activity, dietary magnesium intake, and chronic conditions such as coronary heart disease, diabetes, stroke, and hypertension. Smoking status was categorized into never, former, and current smokers based on whether the participants had smoked <100 cigarettes in their lifetime and their current smoking status. Adequate physical activity was defined as: 75 min per week of vigorous-intensity aerobic activity or at least 150 min per week of moderate-intensity (25). Otherwise, physical activity was inadequate. Magnesium intake was collected from the total nutrient intake provided by the first 24-h dietary recall interview obtained from the Mobile Examination Center. Diabetes was determined by: (1) self-report or medication use; (2) HbA1c level ≥ 6.5%; (3) fasting blood sugar level ≥ 7.0 mmol/L; or (4) 2-h plasma glucose ≥200 mg/dL (26). Hypertension was determined by: (1) self-report or medication use and (2) the average of three systolic blood pressure readings ≥140 mmHg or diastolic blood pressure readings ≥90 mmHg (27). Coronary heart disease and stroke were determined based on self-reported information.

### 2.5 Statistical analysis

Continuous variables were presented as weighted medians and interquartile ranges or weighted means and standard deviations, whereas categorical variables were expressed as frequencies and weight percentages. To detect differences in baseline characteristics between the gout and control groups, we compared continuous variables using Student’s *t*-test and categorical variables using Chi-square test. Multivariable adjusted logistic regression and subgroup analyses were used to assess the relationship between MDS and gout. Confounders were selected based on previous research or a change-in-effect estimate of >10%. Three models were assessed: Model 1 was unadjusted; Model 2 included sex and age; and Model 3 was additionally adjusted for race, smoking status, educational attainment, physical activity, BMI, poverty income ratio, dietary magnesium intake, coronary heart disease, diabetes, stroke, and hypertension. All analyses took into account the complex survey design and incorporated sample weights. Sensitivity analysis was performed using complete case analysis, supplemented by multiple imputations (five iterations) to address any missing data. The results were presented as odds ratios with 95% confidence intervals (CIs). A *p* value <0.05 was considered to indicate statistically significant differences. All data computations were performed using R 4.2.2 and Free Statistics software version 2.0 (28).

## 3 Results

### 3.1 Baseline characteristics

In total, 18,039 participants were included in this study, with 851 and 17,188 participants in the gout and control groups, respectively. The two groups exhibited significant differences in baseline characteristics including age, sex, race, BMI, marital status, physical activity, smoking status, MDS, magnesium intake, and comorbidities (Table 1). Participants with gout were older, more likely to be male, married or living with a partner, have ever smoked, be physically active, and have a higher MDS compared with participants without gout. In addition, participants with gout had a higher risk of diabetes, stroke, hypertension, and coronary heart disease than those without gout did.

**Table 1:** Participants characteristics, weighted.

Variables	Total	With gout	Without gout	*p* value
	*n* = 18,039	*n* = 851	*n* = 17,188	
Age, year	46.96 (16.48)	60.05 (13.08)	46.41 (16.38)	<0.001
Sex, male	9,541 (51.18)	646 (73.53)	8,895 (50.25)	<0.001
BMI, kg/m^2^	29.0 (6.77)	32.0 (6.88)	28.9 (6.74)	<0.001
Race				<0.001
Mexican American	2,600 (7.66)	52 (2.73)	2,548 (7.86)	
Other Hispanic	1,783 (4.96)	49 (2.14)	1,734 (5.08)	
Non-Hispanic White	8,552 (71.76)	468 (78.04)	8,084 (71.50)	
Non-Hispanic Black	3,538 (9.62)	214 (11.23)	3,324 (9.55)	
Other races	1,566 (6.00)	68 (5.86)	1,489 (6.00)	
Education level, year				0.246
<9	3,989 (14.47)	198 (15.13)	3,791 (14.44)	
9–12	4,097 (21.62)	227 (24.25)	3,870 (21.51)	
>12	9,953 (63.92)	426 (60.62)	9,527 (64.06)	
Marital status				<0.001
Married or living with a partner	10,842 (64.42)	572 (73.65)	10,270 (64.04)	
Widowed/ divorced/ separated	3,896 (17.85)	218 (19.34)	3,678 (17.79)	
Never married	3,301 (17.73)	61 (7.00)	3,240 (18.17)	
Poverty income ratio	3.17 (1.57, 5.00)	3.1 (1.57, 5.00)	3.1 (1.57, 5.00)	0.852
Physical activity				<0.001
Inadequate	10,265 (61.71)	397 (52.54)	9,868 (62.09)	
Adequate	7,774 (38.29)	454 (47.46)	7,320 (37.91)	
Smoking status				<0.001
Never	8,942 (51.18)	324 (40.43)	8,618 (51.63)	
Former	4,919 (27.25)	375 (43.17)	4,544 (26.59)	
Current	4,178 (21.57)	152 (16.40)	4,026 (21.78)	
MDS				<0.001
0	4,871 (26.26)	88 (12.28)	4,783 (26.88)	
1	8,606 (49.64)	293 (40.00)	8,313 (50.04)	
2	3,299 (18.21)	281 (30.42)	3,018 (17.70)	
≥3	1,263 (5.86)	189 (17.30)	1,074 (5.38)	
Dietary magnesium intake, mg	273.0 (198.0, 371.0)	265.0 (186.0, 354.5)	273.0 (198.0, 372.0)	0.003
Hypertension	7,472 (36.87)	681 (76.27)	6,791 (35.23)	<0.001
Diabetes	3,183 (13.21)	334 (33.43)	2,849 (12.37)	<0.001
Coronary heart disease	741 (3.37)	122 (11.58)	619 (3.03)	<0.001
Stroke	633 (2.59)	85 (7.35)	548 (2.39)	<0.001

BMI, Body mass index; MDS, Magnesium depletion score. Data are presented as unweighted number (weight percentage) for categorical variables, weighted mean (standard deviation), or weighted median (interquartile range) for continuous variables.

### 3.2 Relationship between MDS and gout

Table 2 shows the association between MDS and gout risk using weighted multivariable logistic regression. After adjusting for confounders, individuals with MDS 1, 2, and ≥ 3 had a 1.45- (95% CI, 0.93–2.26), 1.86- (95% CI, 1.18–2.93), and 2.17-fold (95% CI, 1.37–3.43) increased risk of gout, respectively, compared with participants with an MDS of 0 (*p* for trend <0.001). Thus, adults with a higher MDS were more likely to develop gout.

**Table 2:** Multivariable logistics regression analysis of the association between magnesium depletion score and gout, weighted.

Variable	Unweighted participants	Unadjusted model		Model 1^a^		Model 2^b^		Model 3^c^	
	Event, *n* (%)	OR (95% CI)	*p* value	OR (95% CI)	*p* value	OR (95% CI)	*p* value	OR (95% CI)	*p* value
MDS = 0	88/4871 (1.8)	1 (Reference)		1 (Reference)		1 (Reference)		1 (Reference)	
MDS = 1	293/8606 (3.4)	1.75 (1.16 ~ 2.65)	0.009	1.43 (0.93 ~ 2.20)	0.105	1.48 (0.96 ~ 2.30)	0.077	1.45 (0.93 ~ 2.26)	0.103
MDS = 2	281/3299 (8.5)	3.76 (2.52 ~ 5.61)	<0.001	2.14 (1.38 ~ 3.33)	<0.001	2.09 (1.34 ~ 3.26)	0.002	1.86 (1.18 ~ 2.93)	0.008
MDS ≥ 3	189/1263 (15.0)	7.04 (4.69 ~ 10.57)	<0.001	3.07 (1.97 ~ 4.80)	<0.001	2.73 (1.73 ~ 4.30)	<0.001	2.17 (1.37 ~ 3.43)	0.001
*p* for trend			<0.001		<0.001		<0.001		<0.001

MDS, Magnesium depletion score; BMI, Body mass index. Unadjusted model: no other covariates are adjusted.

a Adjusted by sex and age.

b Adjusted for Model 1+ race, marital status, smoking status, education level, physical activity, BMI, and poverty income ratio.

c Adjusted for Model 2 + dietary magnesium intake, coronary heart disease, diabetes, stroke, and hypertension.

### 3.3 Sensitivity analysis

**Figure 2:**
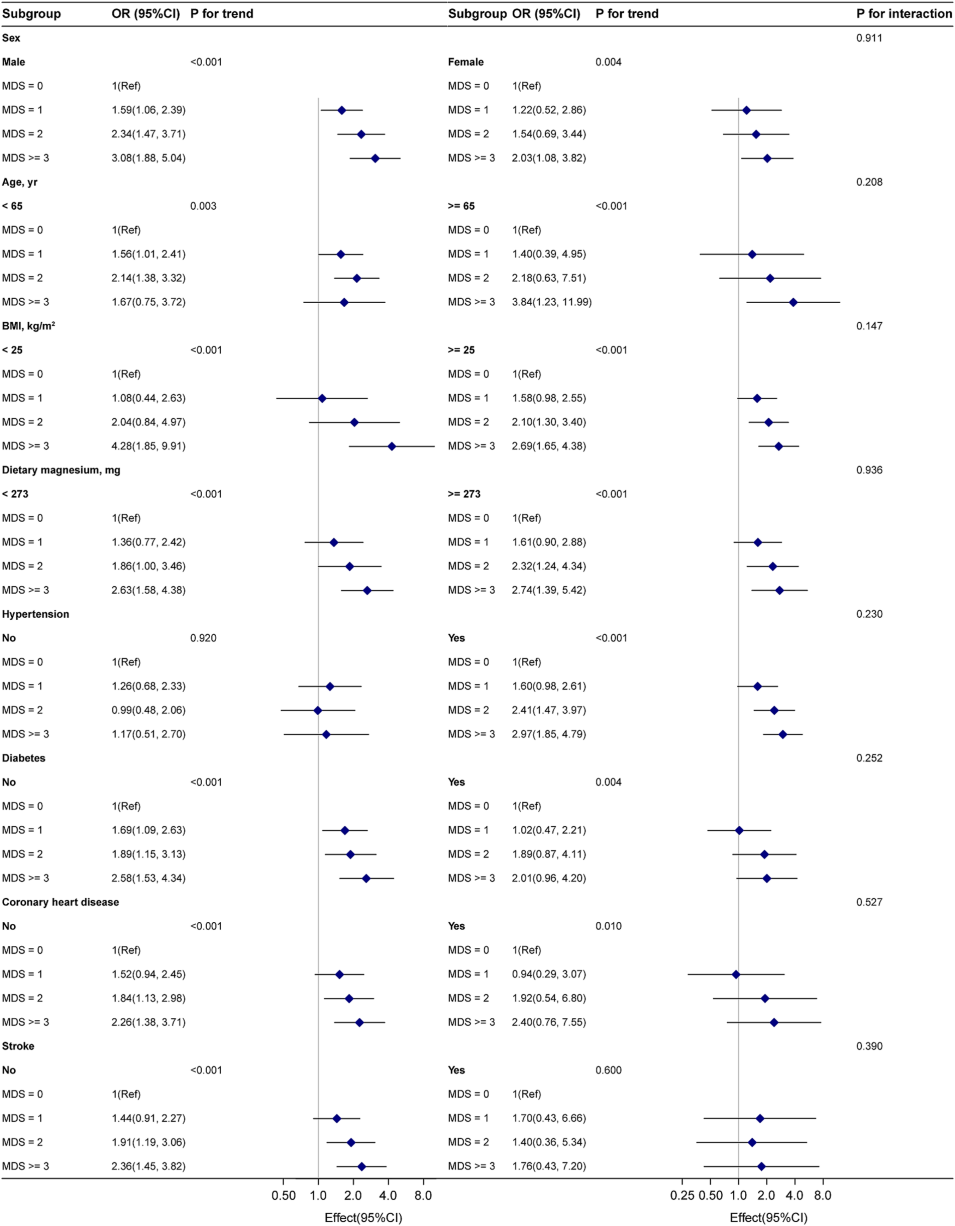
Subgroup analyses of MDS and gout. Adjustment by sex, age, BMI, race, smoking status, education level, physical activity, poverty income ratio, dietary magnesium intake, coronary heart disease, diabetes, stroke, and hypertension.

To evaluate the stability of the association between MDS and gout within various subgroups, we conducted a subgroup analysis. Interaction tests indicated no statistically significant differences in the association between MDS and gout among different subgroups (Figure 2, *p* for interaction >0.05), suggesting that these factors did not significantly influence this positive association. Sensitivity analysis revealed that the relationship between MDS and gout was not significantly affected by multiple imputations of missing data (Supplementary Table S1).

## 4 Discussion

We utilized NHANES data from five cycles (2007–2016) to investigate the association between MDS and gout risk in adults in the United States. The estimated overall prevalence of gout among adults in the United States during this period was 4.7%. After adjusting for confounders, we observed a significant positive correlation between MDS and gout risk. Subgroup analysis confirmed the stability of this association. Dietary magnesium intake did not moderate the positive correlation between MDS and gout risk.

Individuals with normal serum magnesium levels may experience magnesium deficiencies and respond to supplementation (18). The magnesium tolerance test is more accurate but impractical because of the need for 24-h urine collection and intravenous infusion (29). Fan et al. developed an MDS by combining four factors (alcohol consumption, PPI use, diuretic use, and chronic kidney disease) and validated its accuracy compared with that of the magnesium tolerance test (17). The evaluated factors reduce magnesium reabsorption. Alcohol consumption leads to rapid magnesium excretion in the urine (30). Loop-blocking diuretics cause magnesium loss by altering the renin-angiotensin-aldosterone system as well as calcium and parathyroid hormone concentrations (31). PPIs reduce renal magnesium reabsorption by downregulating the activity of the epithelial magnesium channel transient receptor potential melastatin 6 (32, 33). Under certain pathological conditions such as chronic kidney disease, large changes in renal magnesium reabsorption can occur. Chronic kidney disease is associated with increased urinary magnesium excretion (16).

Magnesium has unique antioxidant and anti-inflammatory properties. Research suggests that increased dietary magnesium intake is associated with a reduced risk of hyperuricemia (34), prompting the exploration of its interaction with MDS in gout risk. Our results confirm that dietary magnesium intake does not moderate the correlation between MDS and gout incidence. This finding aligns with those of previous studies, indicating a minimal impact of dietary magnesium on the association between MDS and chronic obstructive pulmonary disease (35), congestive heart failure (36), and cardiovascular diseases (18). Our results underscore the importance of considering the systemic magnesium status in gout risk reduction. When the magnesium status is suboptimal, relying solely on dietary intake may not effectively mitigate the risk of gout, similar to in other related diseases.

Given that gout patients often present with hyperuricemia and both acute or chronic inflammation (1), we hypothesize that magnesium deficiency, as indicated by MDS, may contribute to gout development through these pathways. Firstly, magnesium is involved in a wide range of biochemical reactions, particularly intracellular phosphorylation, which is crucial for initiating DNA synthesis and cell proliferation (37, 38). Additionally, low magnesium levels can affect oxidative stress, leading to oxidative DNA modifications and impaired DNA repair (39). This may result in significant DNA damage and the release of purine nucleotides, whose catabolism ultimately produces uric acid (40). Secondly, experimental studies have shown that magnesium deficiency is associated with acute inflammatory responses mediated by calcium, N-methyl-D-aspartate, and tumor necrosis factor-alpha (41), along with increases in C-reactive protein, interleukin-6, and fibrinogen (42–44), all of which are sensitive biomarkers of inflammation. Although further mechanistic studies are needed, the available evidence partially supports the possibility of the proposed mechanism.

Our research has found that high MDS significantly increases the risk of gout among American adults. Therefore, addressing the four key factors involved in MDS—alcohol consumption, PPI use, diuretic use, and chronic kidney disease—by adopting strategies to reduce magnesium depletion could help lower the risk of gout. Specific measures include reducing alcohol intake, appropriately using PPIs and diuretics, and managing chronic kidney disease. Reducing alcohol consumption can lower urinary magnesium excretion (45); PPIs are commonly prescribed for gastrointestinal issues, but their overuse has exceeded the actual number of necessary cases (46–48); diuretics are used to manage conditions such as hypertension and heart failure, but they can also lead to magnesium loss in urine, highlighting the risk of overuse (49). Monitoring and regulating the use of PPIs and diuretics is crucial, and healthcare providers should balance the benefits of these treatments against the potential risk of magnesium depletion. Lastly, CKD is a progressive, incurable condition related to magnesium metabolism abnormalities (50). Patients with CKD need to be particularly cautious about alcohol and medication usage to avoid exacerbating magnesium depletion, which could increase the risk of gout.

This study had some limitations. First, as a cross-sectional study, it inherently cannot establish causality. Although the association between MDS and gout may be explained by hyperuricemia and inflammatory mechanisms, further mechanistic studies are required to confirm this hypothesis. Second, cross-sectional studies inherently face issues with missing data. We addressed this by performing multiple imputations for missing covariate data, and the results remained stable. Third, gout data from self-reports may have been affected by recall bias. Fourth, dietary magnesium intake was assessed through a single 24-h recall, which may not accurately reflect long-term dietary habits or magnesium status over time. Fifth, while our results are based on NHANES, which uses a complex multistage probability sampling design to obtain a nationally representative sample of non-institutionalized United States adults, the generalizability of these findings to other geographic regions or racial/ethnic groups requires further validation. Finally, although we adjusted for some confounders, other factors such as genetics, lifestyle, and environment may have influenced the results.

## 5 Conclusion

According to the MDS, a positive correlation exists between magnesium deficiency and gout risk among adults in the United States. Additionally, dietary magnesium intake did not alter this association.

## Data Availability

The datasets presented in this study can be found in online repositories. The names of the repository/repositories and accession number(s) can be found at: NHANES is a public dataset that can be freely accessed at http://www.cdc.gov/nchs/nhanes/.
