# Supplementary material for: Magnesium depletion score and gout: insights from NHANES data
Source: Front Nutr. 2024 Nov 21;11:1485578. doi: 10.3389/fnut.2024.1485578 (PMC11617175; doi:10.3389/fnut.2024.1485578)
Supplement: Supplementary file 1 [file Table_1.docx]

Table S1. Multivariable logistics regression analysis of the association between magnesium depletion score and gout by multiple imputations of missing datasets, weighted.

| Variable | Unweighted participants | Unadjusted model | |  | Model 1^a^ | |  | Model 2^b^ | | Model 3^c^ | |
| --- | --- | --- | --- | --- | --- | --- | --- | --- | --- | --- | --- |
|  | Event, n (%) | OR, 95% CI | *P*-value |  | OR, 95% CI | *P*-value |  | OR, 95% CI | *P*-value | OR, 95% CI | *P*-value |
| MDS = 0 | 100/5477 (1.8) | 1 (Reference) |  |  | 1 (Reference) |  |  | 1 (Reference) |  | 1 (Reference) |  |
| MDS = 1 | 316/9692 (3.3) | 1.62 (1.12~2.35) | 0.011 |  | 1.31 (0.90~1.92) | 0.160 |  | 1.37(0.93~2.01) | 0.112 | 1.33 (0.89~1.98) | 0.155 |
| MDS = 2 | 322/3750 (8.6) | 3.72 (2.64~5.26) | <0.001 |  | 2.07 (1.42~3.02) | <0.001 |  | 2.03(1.39~2.96) | <0.001 | 1.80 (1.22~2.66) | 0.004 |
| MDS ≥ 3 | 232/1474 (15.7) | 7.10 (4.95~10.18) | <0.001 |  | 2.99 (2.04~4.39) | <0.001 |  | 2.64(1.77~3.91) | <0.001 | 2.09 (1.42~3.12) | <0.001 |
| *P* for trend |  |  | <0.001 |  |  | <0.001 |  |  | <0.001 |  | <0.001 |

MDS, magnesium depletion score; BMI, body mass index.

Unadjusted model: no other covariates are adjusted;

^a^Adjusted by sex and age; ^b^Adjusted for Model 1+ race, marital status, smoking status, education level, physical activity, BMI, poverty income ratio; ^c^Adjusted for Model 2 + dietary magnesium intake, coronary heart disease, diabetes, stroke, and hypertension.
